# Supplementary material for: Causal Relationship between Aging and Anorexia Nervosa: A White-Matter-Microstructure-Mediated Mendelian Randomization Analysis
Source: Biomedicines. 2024 Aug 16;12(8):1874. doi: 10.3390/biomedicines12081874 (PMC11351342; doi:10.3390/biomedicines12081874)
Supplement: Supplementary file 1 [file biomedicines-12-01874-s001.zip › Supplementary Figure/Figure S4/Singe SNP plot of MR analysis for TL to ubm-b-1928.pdf]

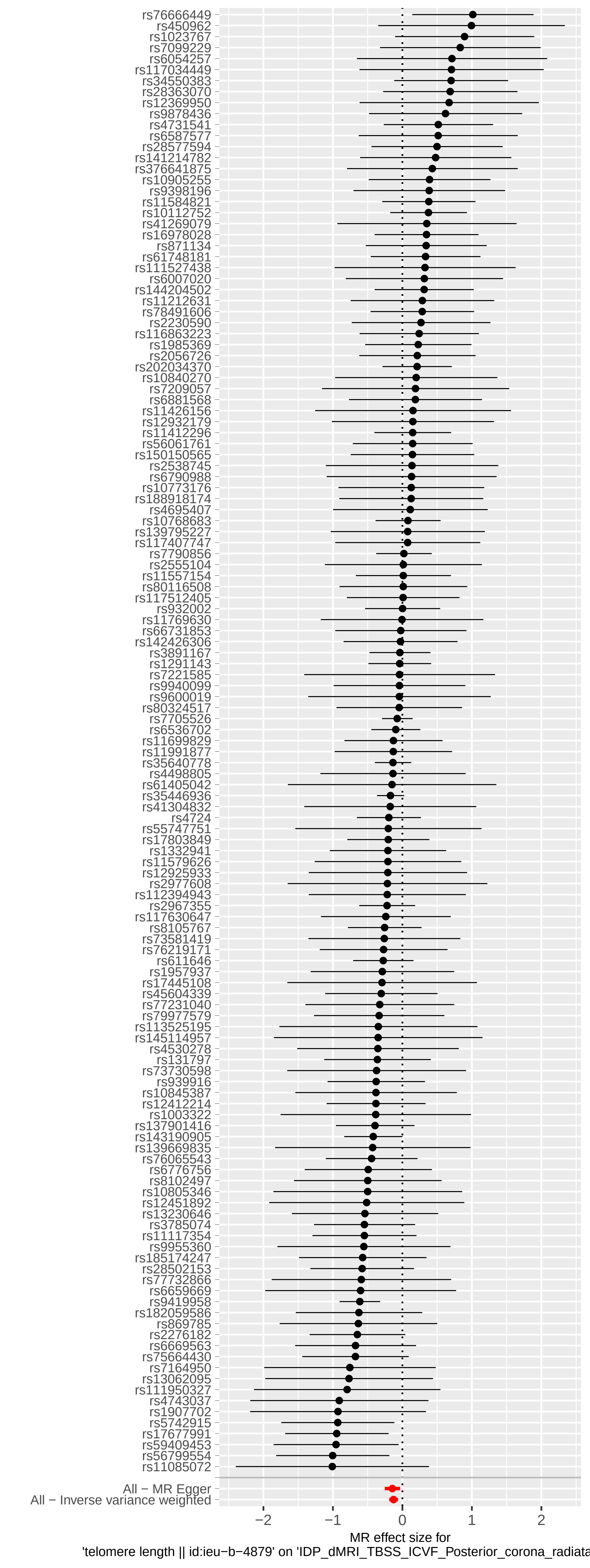
rs76666449
rs450962
rs1023767
rs7099229
rs6054257
rs117034449
rs34550383
rs28363070
rs12369950
rs9878436
rs4731541
rs6587577
rs28577594
rs141214782
rs376641875
rs10905255
rs9398196
rs11584821
rs10112752
rs41269079
rs16978028
rs871134
rs61748181
rs111527438
rs6007020
rs144204502
rs11212631
rs78491606
rs2230590
rs116863223
rs1985369
rs2056726
rs202034370
rs10840270
rs7209057
rs6881568
rs11426156
rs12932179
rs11412296
rs56061761
rs150150565
rs2538745
rs6790988
rs10773176
rs188918174
rs4695407
rs10768683
rs139795227
rs117407747
rs7790856
rs2555104
rs11557154
rs80116508
rs117512405
rs932002
rs11769630
rs66731853
rs142426306
rs3891167
rs1291143
rs7221585
rs9940099
rs9600019
rs80324517
rs7705526
rs6536702
rs11699829
rs11991877
rs35640778
rs4498805
rs61405042
rs35446936
rs41304832
rs4724
rs55747751
rs17803849
rs1332941
rs11579626
rs12925933
rs2977608
rs112394943
rs2967355
rs117630647
rs8105767
rs73581419
rs76219171
rs611646
rs1957937
rs17445108
rs45604339
rs77231040
rs79977579
rs113525195
rs145114957
rs4530278
rs131797
rs73730598
rs939916
rs10845387
rs12412214
rs1003322
rs137901416
rs143190905
rs139669835
rs76065543
rs6776756
rs8102497
rs10805346
rs12451892
rs13230646
rs3785074
rs11117354
rs9955360
rs185174247
rs28502153
rs77732866
rs6659669
rs9419958
rs182059586
rs869785
rs2276182
rs6669563
rs75664430
rs7164950
rs13062095
rs111950327
rs4743037
rs1907702
rs5742915
rs17677991
rs59409453
rs56799554
rs11085072
All − MR Egger
All − Inverse variance weighted
−2
−1
0
1
2
MR effect size for
'telomere length || id:ieu−b−4879' on 'IDP_dMRI_TBSS_ICVF_Posterior_corona_radiata
